# Supplementary material for: Docosahexaenoic Acid as the Bidirectional Biomarker of Dietary and Metabolic Risk Patterns in Chinese Children: A Comparison with Plasma and Erythrocyte
Source: Nutrients. 2022 Jul 28;14(15):3095. doi: 10.3390/nu14153095 (PMC9370652; doi:10.3390/nu14153095)
Supplement: Supplementary file 1 [file nutrients-14-03095-s001.zip › nutrients-1827144-supplementary.pdf]

Table S1. Factor loadings for dietary patterns of children

Food items	Balance pattern	Plant pattern	Beverage & snacks pattern
Rice	0.322	0.189	-0.005
Coarse cereals	0.198	0.483	0.195
Wheat floor	0.218	0.333	0.106
Tubers	0.433	0.340	-0.260
Soybean and its products	0.383	0.433	0.261
Fresh beans	0.453	0.232	0.099
Leaf-off vegetable	0.544	0.388	-0.214
Leafy vegetable	0.176	-0.203	0.021
Fruits	0.591	-0.005	0.009
Meat	0.397	-0.460	-0.006
Poultry	0.510	-0.327	0.106
Fish	0.411	-0.240	-0.332
Shrimp, crab and shellfish	0.315	-0.123	-0.332
Egg	0.224	-0.273	-0.174
Milk and its products	0.207	-0.174	0.384
Nuts	0.553	-0.173	-0.041
Fungi and algae	0.490	-0.067	-0.293
Snacks	0.343	-0.205	0.535
Beverage	0.268	-0.138	0.548
